# Supplementary material for: miR-1233-3p Inhibits Angiopoietin-1-Induced Endothelial Cell Survival, Migration, and Differentiation
Source: Cells. 2025 Jan 8;14(2):75. doi: 10.3390/cells14020075 (PMC11763389; doi:10.3390/cells14020075)
Supplement: Supplementary file 1 [file cells-14-00075-s001.zip › cells-3351002-supplementary.pdf]

Supplementary Materials:

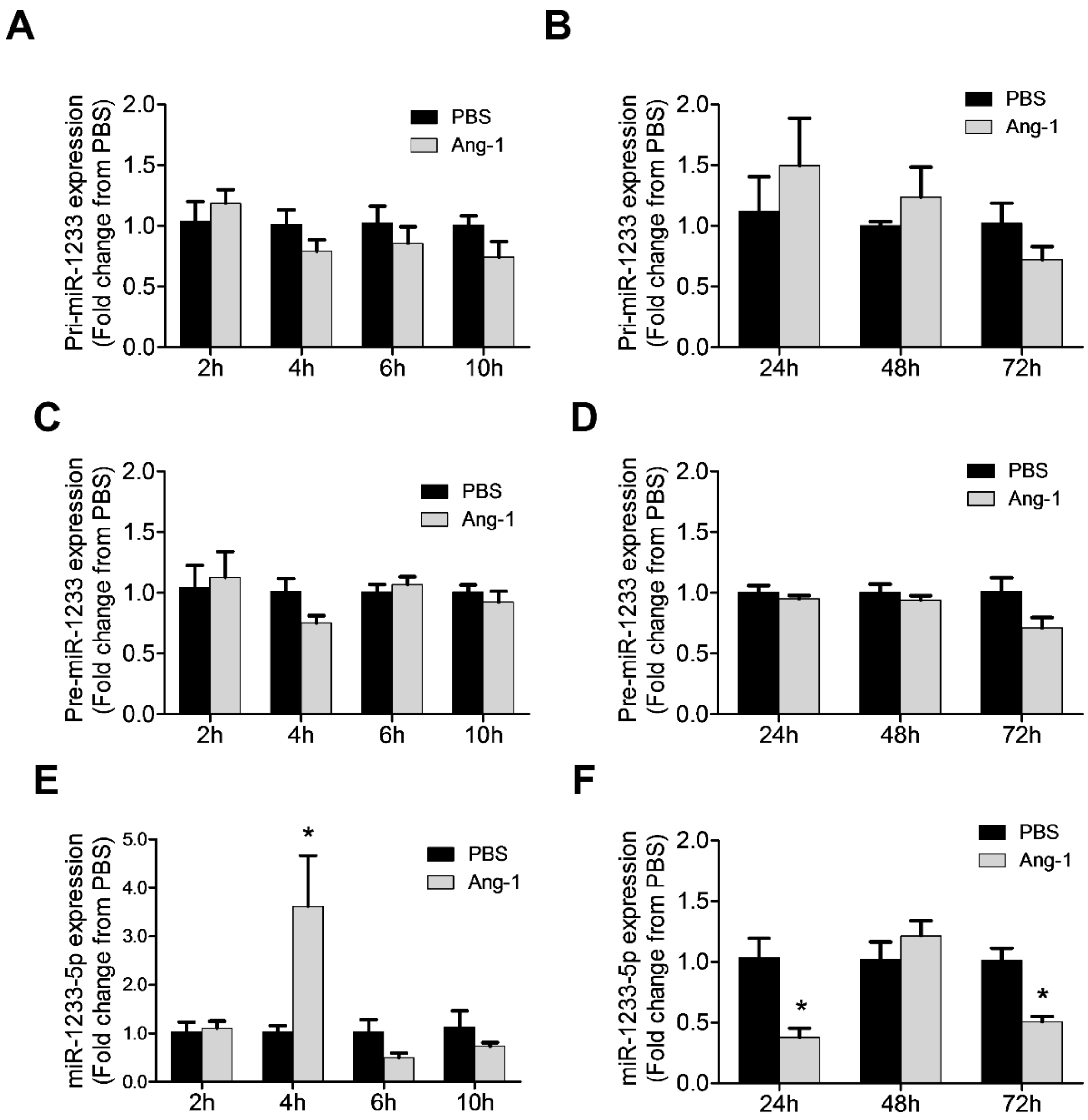

Supplementary figure S1: Ang-1 regulation of pri-miR-1233, pre-miR-1233 and mature miR-1233-5p expressions in HUVECs.

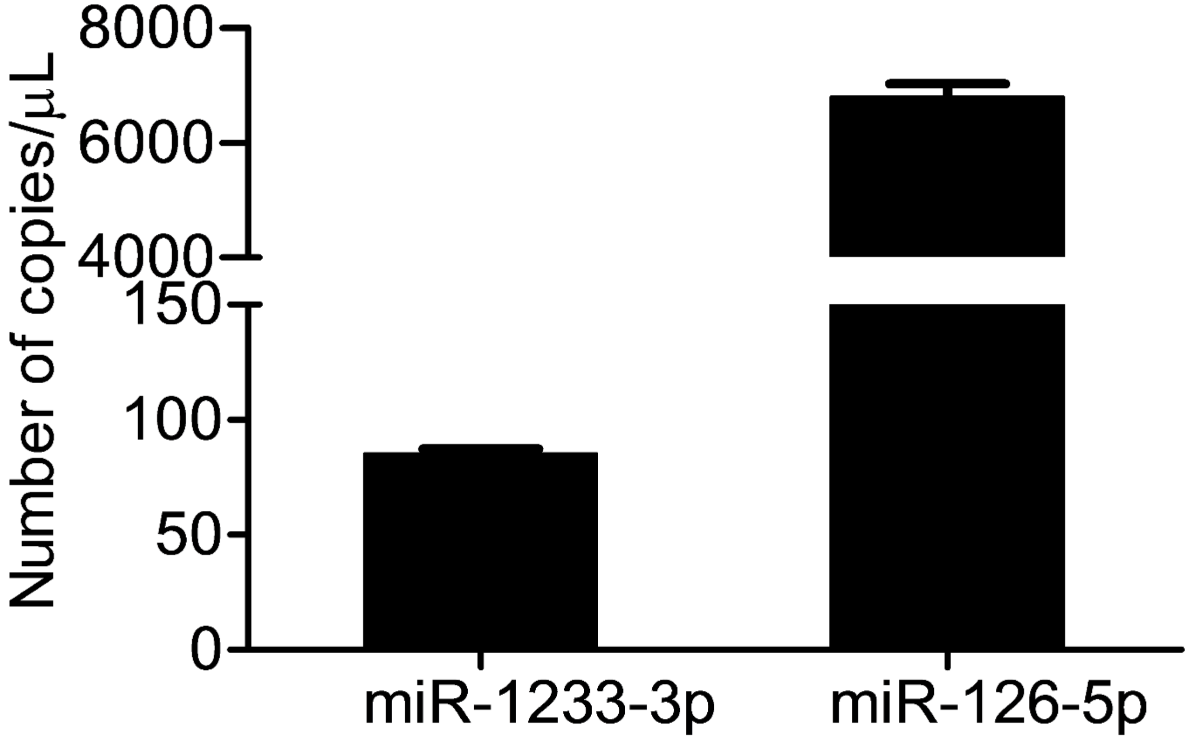

Supplementary figure S2: Abundance of miR-1233-3p in HUVECs

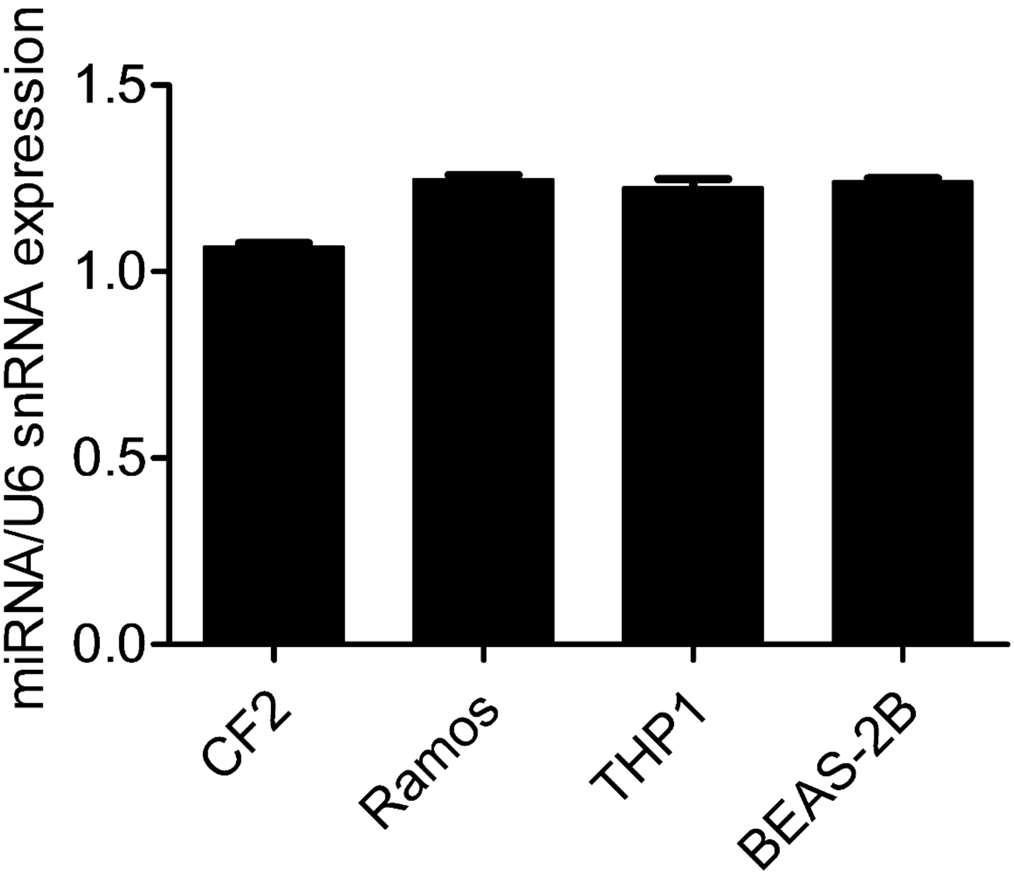

Supplementary figure S3: Detection of miR-1233-3p in different cell types

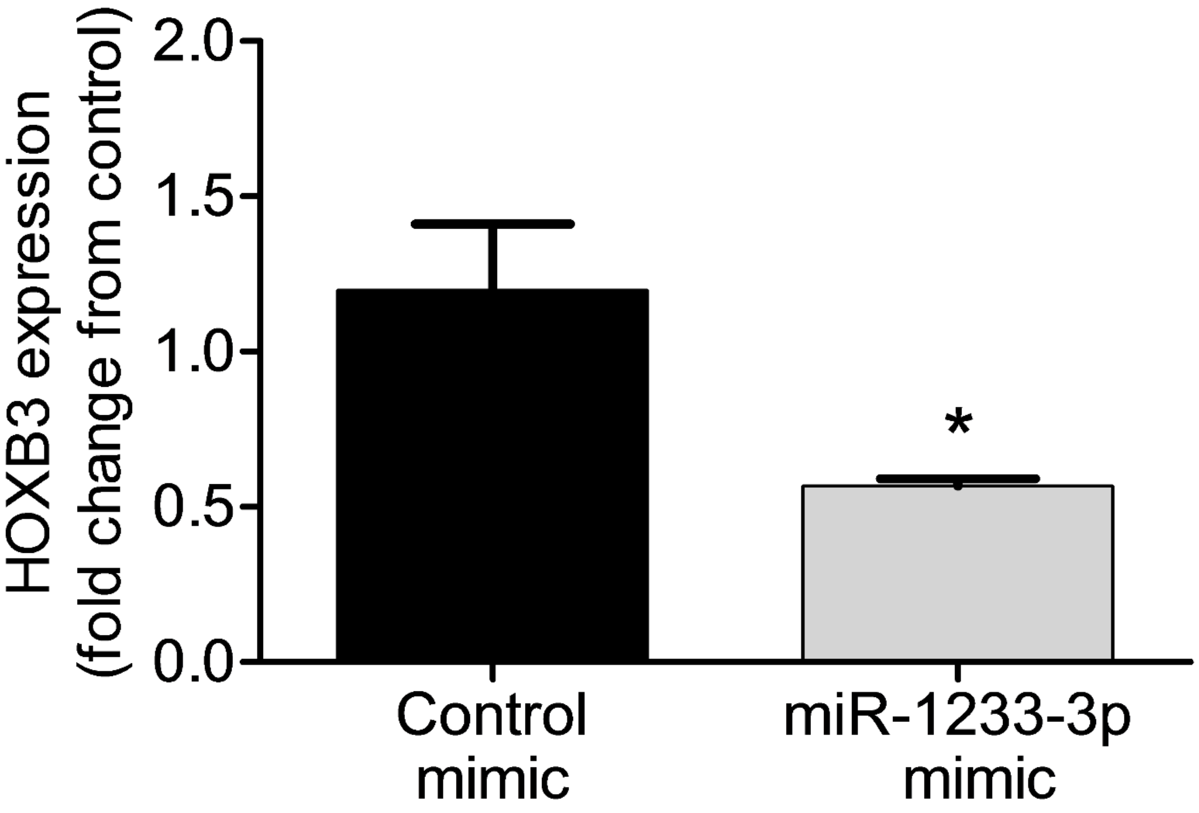

Supplementary figure S4: Transfection efficiency of miR-1233-3p mimics in HUVECs

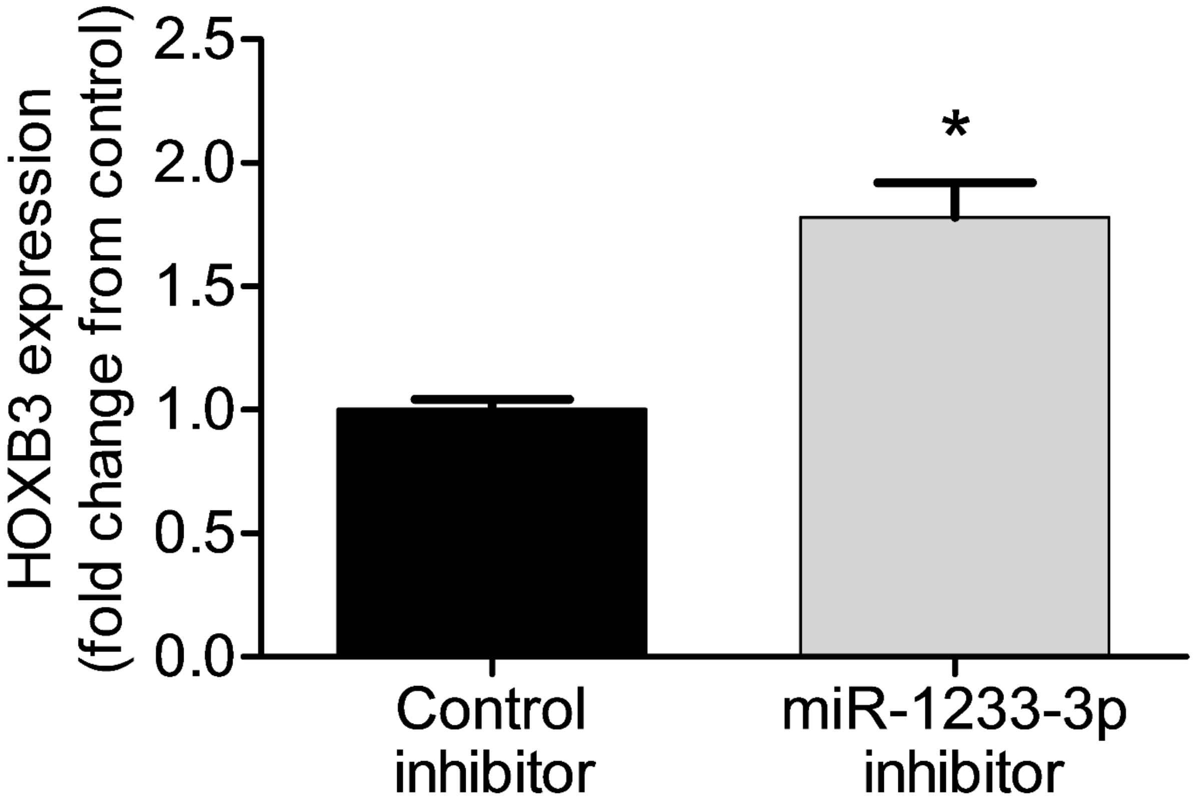

Supplementary figure S5: Transfection efficiency of miR-1233-3p inhibitor in HUVECs.

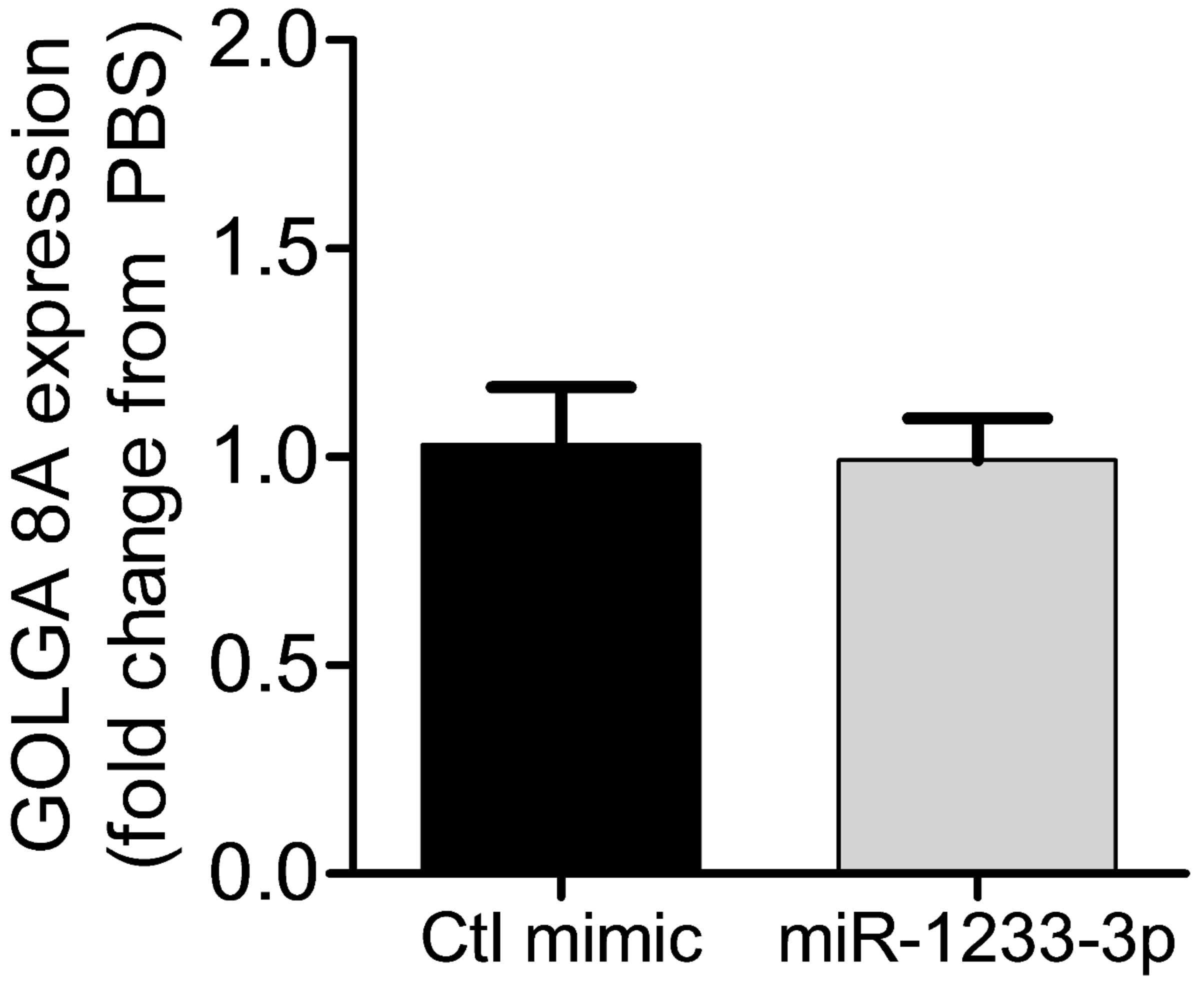

Supplementary figure S6: Effects of miR-1233-3p on GOLGA8A mRNA expressions

Supplementary figure S1: Effects of Ang-1 on pri-miR-1233, pre-miR-1233 and mature miR-1233-5p levels in HUVECs

A-F: A: Levels of pri-miR-1233, pre-miR-1233 and miR-1233-5p in HUVECs exposed for 2, 4, 6, 10, 24, 48 and 72 hours to Ang-1 (300ng/ml) or PBS. Values are expressed as means± SEM and are fold change from PBS. *P<0.05, compared with pre-incubation with PBS.

Supplementary figure S2: Abundance of miR-1233 in HUVECs

Copies of miR-1233-3p and miR-126-5p per µL in HUVECs measured with Droplet digital PCR. Values are expressed as means± SEM.

Supplementary figure S3: Detection of miR-1233-3p in different cell types.

Levels of miR-1233-3p presence in CF2, Ramos, THP1 and BEAS-2B cells measured using qPCR. Calculated as miRNA expression normalized to snoU6 expression. Values are expressed as means± SEM.

Supplementary figure S4: Transfection efficiency of miR-1233-3p mimics in HUVECs

HOXB3 mRNA levels after 48 hours of transfection of control or miR-1233-3p mimics in HUVECs. Values are expressed as means± SEM. *P<0.05 compared to control mimic.

Supplementary figure S5: Transfection efficiency of miR-1233-3p inhibitor in HUVEC.

HOXB3 mRNA levels after 48 hours of transfection of control or miR-1233-3p inhibitors in HUVECs. Values are expressed as means± SEM. *P<0.05 compared to control inhibitor.

Supplementary figure S6: Effects of miR-1233-3p on GOLGA8A mRNA levels

GOLGA8A mRNA levels measured in HUVECs transfected with control or miR-1233-3p mimic mimics. Values are means± SEM and are expressed as fold change from control mimic.
